# Editorial: Cellular and molecular mechanisms of T cell dysfunction: implications for therapy in chronic inflammation

**DOI:** 10.3389/fimmu.2026.1960808

**Published:** 2026-09-03

**Authors:** Sandip Ashok Sonar, Surya Prakash Pandey, Roopesh Singh Gangwar

**Affiliations:** 1 Department of Immunobiology, University of Arizona, Tucson, AZ, United States; 2 University of Arizona College of Medicine, Phoenix, AZ, United States; 3 Department of Immunology, University of Pittsburgh, Pittsburgh, PA, United States; 4 Division of Asthma, Allergy and Immunology, School of Medicine, University of Virginia, Charlottesville, VA, United States

**Keywords:** chronic inflammation, immune regulation, immune-mediated pathologies, regulatory T cells, T cell activation and differentiation, T cell homeostasis

Chronic inflammation is a highly intricate biological process that significantly impacts human health and deteriorates quality of life. It is characterized by persistent dysregulation and activation of various components of the innate and adaptive immune system, as well as non-immune tissue stromal microenvironment. Chronic inflammation not only disrupts tissue homeostasis but also impairs immune regulation, hindering the development of effective protective responses and leading to an increased susceptibility to infections and other inflammatory diseases. Recent research increasingly emphasizes the close interconnection between chronic inflammation and age-related immune dysregulation – broadly referred as immunosenescence (1). Chronic inflammatory conditions and immunosenescence, share multiple common molecular hallmarks and they both are associated with persistent low-level inflammatory state and a decline in tissue and immune homeostasis (2). T lymphocytes (T cells) are essential components of adaptive immunity and play a critical role in immune tolerance, surveillance, pathogen clearance, and anti-tumor immunity. However, chronic inflammation and immunosenescence reprogram T cells, leading to decline in their protective and immunoregulatory functions and acquisition of proinflammatory phenotypes. T cells often exhibit persistent activation in chronic inflammatory conditions, which contributes to tissue damage, exacerbates inflammatory responses, and ultimately drives disease progression. Similarly, aging involves decreased production of new naïve T cells, increased accumulation of memory T cells, and narrowed T cell receptor repertoire. These changes culminate in diminished immune function, ineffective pathogen clearance, and heightened susceptibility to infections and malignancies. Factors such as chronic antigenic stimulation, microenvironmental alterations, dysregulated signaling pathways, and changes in epigenetic regulation collectively contribute to T cell dysfunction and progression of tissue-specific chronic inflammatory diseases (3, 4).

T cell dysfunction involves a broad spectrum of cellular and molecular alterations, including altered differentiation, impaired cytotoxicity, defective memory generation and persistence, imbalanced helper function, metabolic and mitochondrial stress, and epigenetic and transcriptional reprograming (5, 6). Chronic inflammatory conditions such as, autoimmune disease (rheumatoid arthritis, multiple sclerosis, etc.), inflammatory conditions (atherosclerosis, chronic obstructive pulmonary disease, lung fibrosis, liver fibrosis and inflammatory bowel disease) and dysregulated inflammation (during long-term anti-retroviral treatment in HIV patients), all exhibit T cell dysfunction as a common denominator (7, 8). Although substantial progress has been made in defining the phenotypic and functional characteristics of dysfunctional T cell states, the cellular and molecular mechanisms driving the emergence and persistence of these dysfunctional T cells remain poorly understood. The studies included in this Research Topic aimed to address knowledge gap focused on both mouse models of inflammatory conditions as well as human cohort with chronic inflammatory disorders. For example, people living with chronic HIV show immune dysfunction, despite viral suppression and normal CD4 recovery with antiretroviral (ART) therapy. Although ART is highly effective in HIV positive individuals in controlling virus propagation and preventing decline in CD4^+^ T cell counts, it often fails to achieve complete immune restoration and resulted in low CD4/CD8 ratios, a biomarker associated with persistent immune dysfunction and increased morbidity (9). It is well established that T cell metabolism defines their effector immune function (10–12). Garrido-Rodriguez et al. investigated whether metabolic defects contribute to incomplete immune recovery in people living with HIV undergoing ART. The authors identified a metabolic defect in CD8^+^ T cells, characterized by impaired glucose uptake and altered metabolic programming in individual with persistently low (<0.8) CD4/CD8 ratios. These individuals also harbored higher HIV reservoir, as gauged by DNA copy number per million CD4^+^ T cells. These alterations were associated with features of immunosenescence, such as reduced thymic T cell output measured by signal-joint beta T cell receptor excision circle (sj/β TREC), shorter telomere lengths in T cells, and maintenance of viral reservoirs. Whereas people with lower nadir (350 or less) had metabolically active CD8^+^ T cells with no measurable increase in viral reservoir capacity. These results suggest that distinct immunometabolic states may underlie heterogeneous patterns of immune recovery despite successful ART treatment. Further, this study indicates that stratifying people living with HIV based on their CD4/CD8 ratio or nadir score may help understand specific defects in T cell dysfunction.

To elucidate the role of T-cell dysfunction and transcriptional reprogramming in the pathogenesis of COPD, Liu et al. employed single cell transcriptomic profiling with T-cell receptor (TCR) analyses. Their findings provided important mechanistic insights into disease-associated alterations in T-cell populations. COPD is characterized by persistent airway inflammation and progressive lung tissue damage. Dysregulation of a variety of immune cells, including T cells, is implicated in COPD immunopathogenesis. However, until now the molecular signature of T cells and other innate immune cells in the lung and peripheral blood of COPD patients has remained poorly explored. The authors identified that in COPD patients, CD8^+^ T cells exhibit reduced TCR diversity and increased exhausted phenotype with diminished cytotoxic function. The authors further demonstrated that monocyte-derived alveolar macrophages underwent metabolic reprogramming and exhibited a shift to an anti-inflammatory phenotype with reduced phagocytosis and protease-antiprotease imbalance in COPD airways. Further, these metabolically altered macrophages showed increased communication with dysfunctional T cells through SPP1 and GALECTIN signaling. This study provides a transcriptomic-landscape of immune dysfunction within COPD lung microenvironment and suggests a potential mechanism(s) by which innate immune cells, such as monocyte-derived alveolar macrophages contribute to impairment of T cell responses in COPD.

T cell dysfunction paired with natural killer (NK) cells leads to a rare but life-threatening hyperinflammatory condition – hemophagocytic lymphohistiocytosis (HLH). Although HLH is characterized by a pronounced immune activation of macrophages, cytotoxic CD8^+^ T cells and NK cells (13), the immunophenotype of these cells was not systematically analyzed. Qin et al. systematically immunophenotyped various immune cells from the peripheral blood of 75 patients with adult HLH. The authors demonstrated broad immune dysregulation, with altered exhaustion and activation profiles of cytotoxic CD8^+^ T cells and NK cells, indicating that excessive lymphocyte activation can contribute to immune dysfunction. Specifically, reductions in Tregs, central memory and terminal effector memory (TEMRA) T cell populations, and CD56^dim^CD16^+^ subset of NK cells while increased CD56^bright^CD16^−^ and CD56^dim^CD16^−^ NK subsets were noted. Both the T cells and NK cells exhibited increased levels of PD-1, TIGIT, and PD-L1 and decreased expression of CD69, HLA-DR, and CD163 activation markers, accompanied with reduced capacity to degranulate and secrete IFN-γ. Further, the authors analyzed Epstein-Barr virus-associated HLH patients, before and after treatment with immune checkpoint blocker sintilimab – an anti-PD1 mAb, and found an increase in CD8^+^ T cells and CD56^dim^CD16^+^ NK cells and decrease in CD56^bright^CD16^-^ NK cells, rescuing the trend observed in HLH patients. Together, these findings indicate the broad dysregulation of CD8^+^ T cell and NK cell composition, activation status, and functional capacity in HLH patients.

T cell dysfunction is also a characteristic feature of the autoimmune blistering skin disease bullous pemphigoid (BP). Wang et al. discussed the potential role of T cell dysfunction in BP pathogenesis. The authors presented evidence suggesting the impairment in regulatory CD4^+^ T cells (Tregs) function in BP lesions. Tregs maintain peripheral tolerance as well as suppress pathogenic inflammatory responses through various mechanisms involving both cell-cell contact and soluble mediators. In patients with BP, both BP lesions and peripheral blood exhibit reduced Treg abundance and impaired suppressive function (14). Such a loss of Treg-mediated control may facilitate the pathogenic Th2, Th17, and T follicular helper (Tfh) responses. Th2-derived cytokines support B-cell activation, antibody class switching, and eosinophilic inflammation, whereas Tfh-derived IL-21 promotes B-cell differentiation and production of pathogenic anti-BP180/BP230 autoantibodies. Collectively, such dysregulation of T cells and antibody responses may contribute to the accumulation of inflammatory cells, basement membrane zone damage, and blister formation, suggesting Treg cell therapies and/or strategies that facilitate their phenotypic and functional stability may provide therapeutic benefits.

T cells also play a key role in the female reproductive system. CD8^+^ T cells actively perform the immune surveillance of female reproductive tract, including uterine endometrium. Endometrial microenvironment plays a critical decisive role in successful embryo implantation. The endometrium is supplied with the immune environment with various immune cell compositions, and about two-thirds of T cells in human endometrium are CD8^+^ T cells (15). Although studies have investigated NK cell and macrophage function in endometrium, whether response from the endometrial immune compartment influences the recurrent implantation failure is not known. Jiang et al. addressed this question through systematically analyzing clinical history of 110 RIF patients with endometrial immune parameters and machine learning algorithms. The study identified that high implantation failure is associated with low endometrial CD8^+^ T cells levels. Using machine learning, the authors demonstrated that endometrial CD8^+^ T cells above 2.0% significantly improve implantation success, potentially mitigating adverse negative effects of immune dysregulation or potentially controlling harmful targets within the microenvironment. This study highlights that CD8^+^ T cells exhibit a context-dependent function in the local endometrial tissue environment, and CD8^+^ T cell levels may serve as a potential biomarker to identify patients at risk of recurrent implantation failure.

In addition to immune cells, tissue stromal cells such as fibroblasts are increasingly recognized as an important regulator of immune microenvironment. Fibroblasts provide both structural support for lymphoid and non-lymphoid organs, and trophic cues required for homeostatic maintenance and function of various immune cells, including T cells. Zhang et al. compiled a literature review describing potential roles that fibroblasts play, and underlying mechanisms modulating tissue microenvironment and immune cell function in various chronic inflammatory conditions and tumor microenvironment. The authors discuss the evidence supporting the idea that fibroblasts serve as active regulators of immune function in various conditions ranging from chronic inflammatory, autoimmune, fibrotic, and tissue repair. Fibroblasts exhibit these diverse functions through highly heterogenous tissue niches supporting various cell-cell interactions, producing cytokines, chemokines, extracellular matrix remodeling factors, metabolites, etc. These diverse mechanisms help fibroblast niches to integrate various signals from tissue microenvironment, and accordingly, modulate immune cells, including T cell recruitment, localization, differentiation, and persistence. The authors further highlighted therapeutic strategies focused on targeting fibroblast niches via direct ablation of pathogenic fibroblast subset with antibody- or complement-mediated cell cytotoxicity or small molecule inhibitors targeting disease-associated fibroblast populations, such as cancer-associated fibroblasts, or modulating fibroblast function.

Collectively, the contributions to this Research Topic “Cellular and molecular mechanisms of T cell dysfunction: implications for therapy in chronic inflammation” highlight the new findings of various context-dependent cellular and molecular mechanisms that contribute to generate and/or maintain T cell dysfunctional states that underlie the chronic inflammatory disease condition. Further, these studies suggest that the underlying mechanisms involved may stem from T-cell intrinsic defects as well as tissue microenvironment dysfunction, including the critical role that structural stromal cells such as fibroblasts, and innate immune cells, like macrophage play in modulating such inflammatory T cell states and disease pathogenesis.
